# Prognostic clinical indexes for prediction of acute gangrenous cholecystitis and acute purulent cholecystitis

**DOI:** 10.1186/s12876-022-02582-6

**Published:** 2022-11-28

**Authors:** Jie Chen, Qi Gao, Xinyu Huang, Yingqi Wang

**Affiliations:** 1 Department of General Surgery, Shanghai Jiaotong University Affiliated Sixth People’s Hospital, 600# Yishan Rd, Xuhui District, Shanghai, 200233 China; 2 Institute of Brain Sciences, Fudan University, 131# Dong’an Road, Xuhui District, Shanghai, 200032 China

**Keywords:** Preoperative prediction, Acute gangrenous cholecystitis, Acute purulent cholecystitis, Acute exacerbation of chronic cholecystitis

## Abstract

**Background:**

Preoperative prediction of severe cholecystitis (SC), including acute gangrenous cholecystitis (AGC) and acute purulent cholecystitis (APC), as opposed to acute exacerbation of chronic cholecystitis (ACC), is of great significance, as SC is associated with high mortality rate.

**Methods:**

In this study, we retrospectively investigated medical records of 114 cholecystitis patients, treated in Shanghai No. 6 People’s Hospital from February 2009 to July 2020. Gallbladder wall thickness (GBWT), indexes of blood routine examination, including white blood cell (WBC), alkaline phosphatase (ALP), the percentage of neutrophil, alanine transaminase (ALT), aspartate aminotransferase (AST), fibrinogen (FIB), gamma-glutamyl transferase, prothrombin time and total bilirubin were evaluated. One-way analysis of variance (ANOVA) was used to evaluate significant differences between a certain kind of SC and ACC to select a prediction index for each kind of SC. Receiver operating characteristic (ROC) curve analysis was conducted to identify the prediction effectiveness of these indexes and their optimal cut-off values.

**Results:**

Higher WBC and lower ALP were associated with AGC diagnosis (*P* < 0.05). Higher percentage of neutrophils was indicative of APC and AGC, while higher GBWT was significantly associated with APC diagnosis (*P* < 0.05) The optimal cut-off values for these indexes were established at 11.1*10^9^/L (OR: 5.333, 95% CI 2.576–10.68, *P* < 0.0001, sensitivity: 72.73%, specificity: 66.67%), 79.75% (OR: 5.735, 95% CI 2.749–12.05, *P* < 0.0001, sensitivity: 77.92%, specificity: 61.9%) and 5.5 mm (OR: 22, 95% CI 4.757–83.42, *P* < 0.0001, sensitivity: 78.57%, specificity: 85.71%), respectively.

**Conclusion:**

We established a predictive model for the differentiations of APC and AGC from ACC using clinical indexes, such as GBWT, the percentage of neutrophil and WBC, and determined cut-off values for these indexes based on ROC curves. Index values exceeding these cut-off values will allow to diagnose patients as APC and AGC, as opposed to a diagnosis of ACC.

## Background

Cholecystitis is a common kind of surgical disease, which can be ascribed to gallbladder stones or inflammation [1, 2]. Rapid diagnosis of different kinds of cholecystitis is of great significance, as severe cholecystitis (SC), such as acute gangrenous cholecystitis (AGC) and acute purulent cholecystitis (APC), are associated with higher mortality rate than acute exacerbation of chronic cholecystitis (ACC) [3]. Patients suffering from SC may require treatment in the intensive care unit (ICU). Thus, quick, simple, and accurate identification of severe cholecystitis will allow timely treatment of SC patients in ICU and may reduce mortality rates, associated with SC. These three types are difficult to distinguish before surgery, and the diagnosis is made based on the postoperative pathology.

White blood cell (WBC) and neutrophil levels are early inflammatory markers that are detected during blood routine examination (BRE) and used for the diagnosis of cholecystitis [4, 5]. Indexes of BRE, especially indexes related to lymphocytes, have been widely applied in research and diagnosis of cholecystitis [6, 7]. In addition, gallbladder wall thickness (GBWT), measured by B-mode ultrasonography, is associated with acute and chronic cholecystitis, and also serves as a potential predictor of cholecystitis [8]. In this retrospective study, we aimed to find out some sensitive and specific indexes for the early prediction of different kinds of cholecystitis based on BRE and B-mode ultrasonography.

## Methods

### Study design and data collection

We retrospectively reviewed data from patients who were admitted either for emergency or elective surgery with different kinds of cholecystitis in Shanghai No. 6 People’s Hospital from February 2009 to July 2020. This study was approved by the ethics committee of Shanghai No. 6 People’s Hospital [Approval No.: 2021-KY-059 (K)]. Medical data of a total of 114 patients were included.

The inclusion criteria were as follows: Patient older than 18 years; Diagnosis of cholecystitis following the revised Tokyo guidelines 2018 [9], including positive Murphy’s syndrome, peritoneal reaction or mass in right hypochondrium, signs of inflammation and data compatible with cholecystitis in B-mode ultrasonography.

The exclusion criteria were as follows: Unavailable or incomplete medical records; No surgical intervention; Malignancies.

The following clinical indexes were evaluated upon admission: right upper quadrant (RUQ) pain, Murphy’s syndrome, body temperature, jaundice, gallbladder stones, diabetes, BRE indexes (see below) and one ultrasonographic index, GBWT. All data of emergency surgery were collected within 24 h, and data of elective surgery were collected within 48 h of admission. They were done at similar intervals for all groups patients.

The thickness of the gall bladder was measured by B-mode ultrasonography at two different locations in longitudinal scan at the level of anterior wall and mean value was calculated. Ultrasound examination was performed by experienced gastroenterology trainees (> 3 years experience) using GE Versana Premier ultrasound system (GE, USA).

BRE indexes included WBC, alkaline phosphatase (ALP), the percentage of neutrophils, alanine transaminase (ALT), aspartate aminotransferase (AST), fibrinogen (FIB), gamma-glutamyltransferase (GGT), prothrombin time (PT), total bilirubin (TB).

Of 114 patients that met the inclusion criteria, 82 patients were admitted from the emergency room (emergency surgery), and 32 patients underwent the elective surgery. The guidelines for selecting treatment were as follows: antibiotic treatment was the first choice for acute cholecystitis. In case of ineffectiveness of the antibiotic treatment, drainage or surgery were performed. In case of drainage contraindication, the surgery was performed directly. Indications for surgery included chronic calculous cholecystitis, chronic atrophic cholecystitis, and multiple episodes of acute cholecystitis. We excluded cases with successful drainage but no secondary operation in our hospital.

There were no cases with drainage failure and subsequent surgery in our hospital, which may be related to the standardization and precision of drainage technology, or the low sample size in our center. All 82 emergency patients in this article had contraindications to percutaneous gallbladder drainage treatment (such as thickening of gallbladder wall, inability to puncture gallbladder wall, abnormal coagulation function, cystic duct stones, gallbladder perforation, etc.) that justified emergency cholecystectomy.

**Fig. 1:**
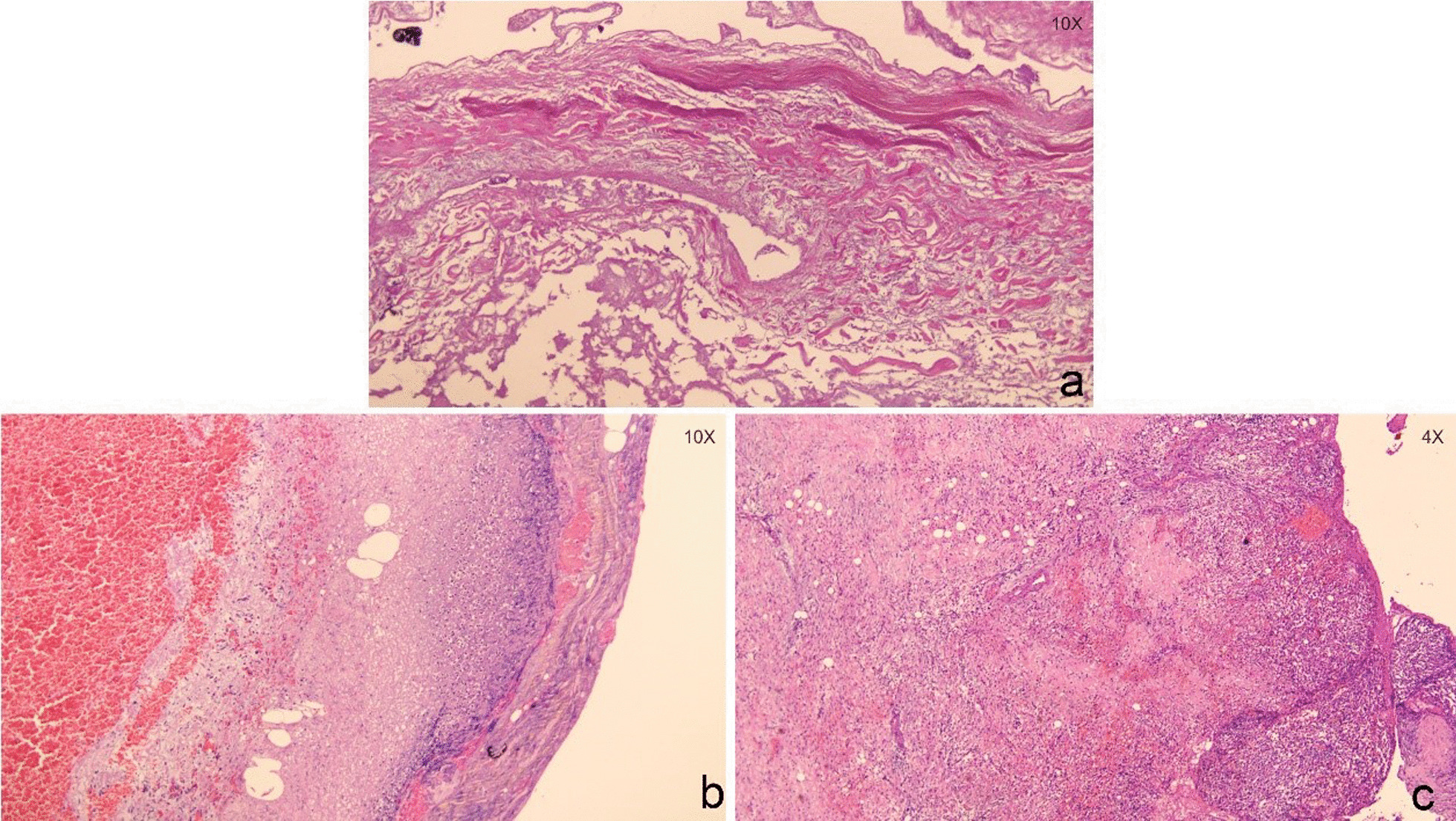
The gallbladder sections of AGC (**a**), APC (**b**) and ACC (**c**)

Laparoscopic cholecystectomy was the first choice of surgery. In cases of severe adhesion to the surrounding tissues due to inflammation and edema, when the Calot triangle was difficult to dissect, laparoscopic procedure was converted into the open surgery. The conversion rate of the patients in the study was 52.94%, with a total of 17 patients undergoing laparoscopic surgery and 97 patients undergoing direct open surgery. Gallbladder tissue was sent for the histopathological examination. Differential diagnosis of AGC, APC and ACC was based on the following parameters:

AGC: The gallbladder wall was in complete disintegration and necrosis status. The structure of the gallbladder wall was fully destroyed. The diseased regions consisted of the whole layers of gallbladder wall (Fig. 1a).

APC: Excessive bleeding was observed on the gallbladder wall. The structure of gallbladder wall was fully destroyed. The necrosis regions consisted of the whole layers of gallbladder wall and the serosal surface. Excessive purulent exudation was observed on the serosal surface (Fig. 1b).

ACC: The gallbladder mucosa fell off. Fibrinoid necrotic exudation was observed on the surface of the gallbladder wall accompanying with infiltrations of neutrophils, gallbladder wall lymphocytes and plasmocytes (Fig. 1c).

Based on the diagnosis, patients were classified as ACC (n = 23), APC (n = 8) and AGC (n = 83) groups. The duration of acute symptoms of AGC was 2.36 days, APC was 2.23 days, and ACC was 1.66 days.

Postoperative complications rate of the patients in the study was 5.26%, including one case of bile leakage, 2 cases of pulmonary infection and 3 cases of incisional infection. The mortality rate was 3.51%.

### Statistical analysis

Data was analyzed using one-way analysis of variance (ANOVA) to determine the differences of a certain index among three kinds of cholecystitis. Once significant differences were detected, we selected this index as a candidate. Receiver operating characteristic (ROC) curve analysis was performed to determine cut-off values for preoperative clinical indexes in the candidate list that could discriminate between ACC and SC. The most prominent point on the ROC curve was chosen as the cut-off value for a certain candidate index. Finally, we investigated the effects of these indexes in the prediction of different kinds of cholecystitis and determined suitable preoperative prediction models for each kind of cholecystitis. Data were described as mean ± standard deviations. Continuous variables were compared using the one-way ANOVA, while categorical variables were compared using the chi-squared test. Multiple regression analyses were performed using a proportional hazards model to identify factors independently associated with three kinds of cholecystectomy, and to estimate corresponding odds ratio (OR) in 95% confidence intervals (CI). Statistical analysis was performed using GraphPad Prism version 7. Statistical significance was accepted for *P*-values less than 0.05.

## Results

### Constitution of study patients

A total of 114 patients, (71 males and 43 females) were included in the study. All patients were retrospectively divided into three groups based on their postoperative pathological diagnosis: acute exacerbation of chronic cholecystitis (ACC, n = 23), acute purulent cholecystitis (APC, n = 8) and acute gangrenous cholecystitis (AGC, n = 83). The rate of emergency/elective surgery of the 3 groups were 35/65%(APC), 84/16%(AGC), and 50/50%(ACC), respectively. As summarized in Table 1, the average age of ACC APC and AGC were respectively 60.64, 58 and 59.60. Among these groups, in terms of comorbidities and systemic diseases, there were 41 cases of hypertension, 13 cases of diabetes, 1 case of bile leakage, 2 cases of pulmonary infection and 3 cases of incisional infection without systemic diseases. The total mortality rate was 3.51%. The average hospital stay of the patients was 9.32 days. 35.65% of patients stayed in ICU after the surgery, with an average of 5.68 days and an average age of 74 years. Among them, 80.49% were emergency patients, and most of them had comorbidities (such as hypertension, pulmonary infection, renal insufficiency, etc.) before the surgery. ACC group was considered as a control group, and the data of all other groups was compared to that of the control group. Among these groups, right upper quadrant (RUQ) pain was the most common symptom, which was observed in 86.19% of patients. The index of Murphy’s syndrome could be applied to effectively distinguish severe types of cholecystitis (APC, AGC) from ACC (Table 1). The duration of acute symptoms of AGC was 2.36 days, APC was 2.23 days, and ACC was 1.66 days.

**Table 1:** Basic clinical characteristics of patients underwent cholecystectomy

Type	Total	Male	Female	RUQ pain (rate%)	P_1_	Murphy’s (rate%)	P_2_
ACC	23	14	9	21 (91.3)	–	4 (17.39)	–
APC	8	3	5	5 (62.50)	0.0564	4 (50.00)	0.0694
AGC	83	55	28	73 (87.95)	0.6534	56 (67.47)	< 0.0001
Total	114	71	43	99 (86.84)	–	64 (56.14)	–

*ACC* Acute exacerbation of chronic cholecystitis; *APC* Acute purulent cholecystitis; *AGC* Acute gangrenous cholecystitis; *RUQ* Right upper quadrant. All indexes are compared to data in ACC group

### Predictive indexes for severe types of cholecystitis

We sought to identify more precise and specific indexes to distinguish different types of SC from ACC. We selected ten types of clinical indexes and compare these indexes between SC and ACC. Using one-way ANOVA, we found that WBC counts and ALP were significantly higher in patients with AGC compared to ACC (15.68*10^9^/L vs. 11.09*10^9^/L, *P* < 0.001). ALP was significantly lower in AGC compared to ACC (102.48 U/L vs. 146.09 U/L, *P* < 0.01). The percentage of neutrophil was significantly different between APC and ACC (85.71% vs. 76.03%, *P* < 0.01) and between AGC and ACC (83.76% vs. 76.03%, *P* < 0.001). The GBWT was significantly higher in APC patients compared to ACC (7.21 mm vs. 4.91 mm, *P* < 0.001). There was no significant difference observed among other groups in terms of other indexes (Table 2).

**Table 2:** Preoperative characteristics of patients underwent cholecystectomy

Parameters	ACC (n = 23) Mean ± SEM / S	AGC (n = 83) Mean ± SEM / P	APC (n = 8) Mean ± SEM / P
WBC (*10^9^/L)	11.09 ± 0.75/–	15.68 ± 0.51/***	14.64 ± 1.65/ns
ALP (U/L)	146.09 ± 20.02/–	102.48 ± 4.71/**	118.79 ± 12.66/ns
N (%)	76.03 ± 1.78/–	83.76 ± 0.80/***	85.71 ± 1.51/**
ALT (U/L)	66.86 ± 16.32/–	45.99 ± 4.27/ns	42.43 ± 4.99/ns
AST (U/L)	49.57 ± 11.00/–	41.60 ± 3.57/ns	38.79 ± 5.83/ns
FIB (g/L)	4.67 ± 0.27/–	4.99 ± 0.14/ns	5.24 ± 0.63/ns
GGT (U/L)	144.57 ± 27.21/–	131.62 ± 16.32/ns	63.79 ± 15.80/ns
PT (s)	12.85 ± 0.26/–	12.73 ± 0.10/ns	13.14 ± 0.41/ns
TB (µmol/L)	34.84 ± 4.77/–	30.82 ± 1.68/ns	28.56 ± 6.55/ns
GBWT (mm)	4.91 ± 0.28/–	4.57 ± 0.11/ns	7.21 ± 0.59/***

*S* Significance; * = *P* < 0.05; ** = *P* < 0.01; *** = *P* < 0.001; *ns* No significance; *ACC* Acute exacerbation of chronic cholecystitis; *APC* Acute purulent cholecystitis; *AGC* Acute gangrenous cholecystitis; *WBC* White blood cell; *ALP* Alkaline phosphatase; *N* Neutrophil; *ALT* Alanine transaminase; *AST* Aspartate aminotransferase; *FIB* Fibrinogen; *GGT* Gamma-glutamyltransferase; *PT* Prothrombin time; *TB* Total bilirubin; *GBWT* Gall bladder wall thickness

### Predictive models for different types of SC

**Fig. 2:**
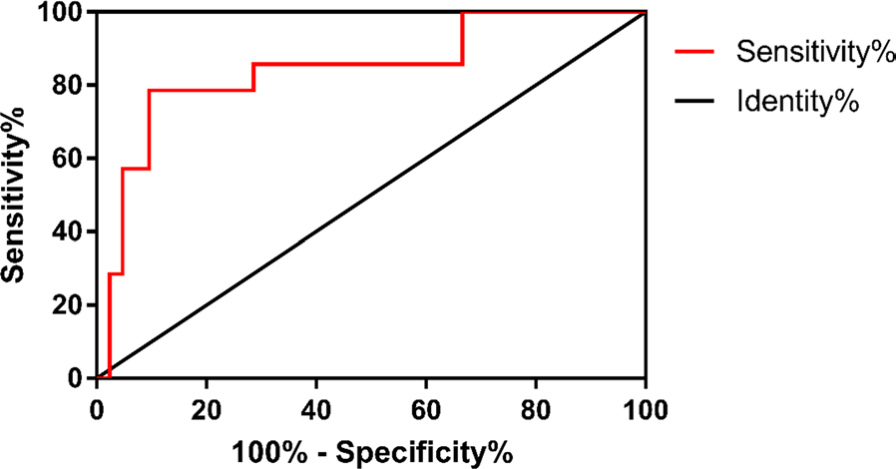
ROC curve of gallbladder wall thickness for acute exacerbation of chronic cholecystitis and acute purulent cholecystitis. The area under curve: 0.8435; 95% CI 0.7118–0.9753; *P* = 0.0001

Based on the above analysis, we next used WBC, ALP, GBWT and the percentage of neutrophil as standard indexes to distinguish AGC and APC from ACC. In order to evaluate the effects of the prediction, we established ROC curves. Considering that significant difference was detected only between APC and ACC in terms of GBWT, it may be a unique index which could be effectively applied to predict APC from ACC (Fig. 2). The result of the GBWT ROC analysis showed that the area under curve (AUC) was 0.8435 (95% CI 0.7118–0.9753, *P* = 0.0001) (Table 3). With a cut-off value of 5.5 mm, the sensitivity was 78.57% and the specificity was 85.71, respectively. The odds ratio (OR) was 22 with statistically significant difference (*P* < 0.0001) (Table 4). Therefore, GBWT could be applied to distinguish APC from ACC reliably.

**Table 3:** ROC analysis for preoperative indexes of patients underwent cholecystectomy

Variables	AUC	95% CI	P
GBWT (APC-ACC)	0.8435	0.7118–0.9753	0.0001
N (AGC-ACC)	0.7073	0.6211–0.7936	< 0.0001
N (APC-ACC)	0.7449	0.6087–0.8811	0.0064
ALP (AGC-ACC)	0.5889	0.4878–0.6900	0.0776
WBC (AGC-ACC)	0.7137	0.6307–0.7966	< 0.0001

*AUC* Area under curve; *CI* Confidence interval; *ACC* Acute exacerbation of chronic cholecystitis; *APC* Acute purulent cholecystitis; *AGC* Acute gangrenous cholecystitis; *WBC* White blood cell; *ALP* Alkaline phosphatase; *N* Neutrophil; *GBWT* Gall bladder wall thickness

**Table 4:** Odds ratio for severe cholecystitis associated with clinical variables in patients underwent cholecystectomy

Variables	GWBT (APC-ACC)	N (AGC-ACC)	WBC (AGC-ACC)	N (APC-ACC)
Cut-off value	5.5 mm	79.75%	11.1*10^9^ /L	80.5%
χ^2^ (1)	20.52	24.64	22.31	9.524
Sensitivity (%)	78.57	77.92	72.73	85.71
Specificity (%)	85.71	61.9	66.67	61.9
Odds ratio	22	5.735	5.333	9.75
95% CI	4.757–83.42	2.749–12.05	2.576–10.68	2.161–46.36
*P*	< 0.0001	< 0.0001	< 0.0001	**0.002**

*GBWT* Gall bladder wall thickness; *N* The percentage of neutrophil; *WBC* White blood cell; *ACC* Acute exacerbation of chronic cholecystitis; *APC* Acute purulent cholecystitis; *AGC* Acute gangrenous cholecystitis

While comparing AGC and ACC in terms of the percentage of neutrophils, we found that the AUC was 0.7073 (95% CI 0.6211–0.7936, *P* < 0.0001) (Table 3). With a cut-off value of 79.75%, the sensitivity was 77.92% and the specificity was 61.9%, respectively. The odds ratio (OR) was 5.735 with statistically significant difference (*P* < 0.0001) (Table 4). Thus, we selected the percentage of neutrophil as a standard index for the prediction and distinguishing of AGC from ACC.

There was no statistically significant difference observed between AGC and ACC (*P* = 0.0776). Thus, ALP was eliminated from the subsequent analysis.

**Fig. 3:**
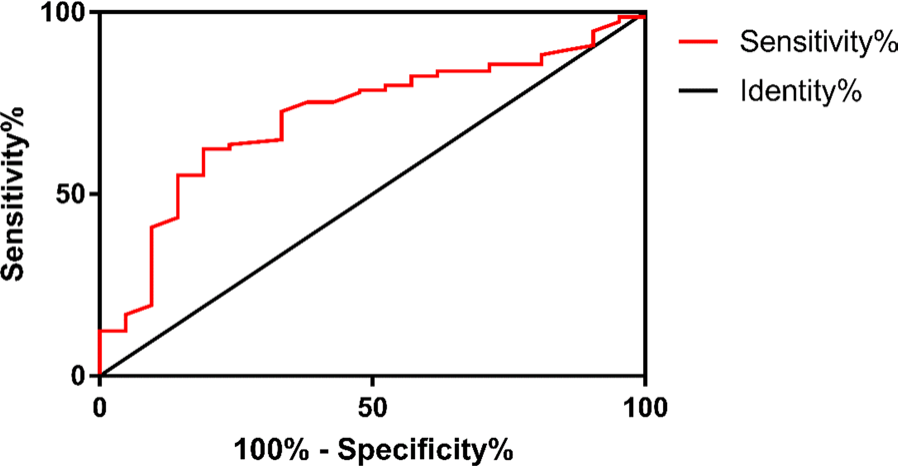
ROC curve of the percentage of white blood cell for acute exacerbation of chronic cholecystitis and acute gangrenous cholecystitis. The area under curve: 0.7137; 95% CI 0.6307–0.7966; *P* < 0.0001

In the analysis of WBC differences between AGC and ACC, we found that the AUC was 0.7137 (95% CI 0.6307–0.7966, *P* < 0.0001) (Fig. 3). With a cut-off value of 11.1*10^9^/L, the sensitivity was 72.73% and the specificity was 66.67%, respectively. The odds ratio (OR) was 5.333 with statistically significant difference (*P* < 0.0001) (Table 4). Thus, WBC could be used as a standard index for the prediction and distinguishing of GC from ACC.

**Fig. 4:**
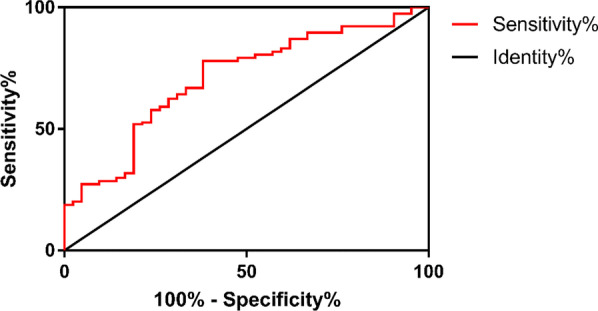
ROC curve of neutrophil counting for acute exacerbation of chronic cholecystitis and acute gangrenous cholecystitis. The area under curve: 0.7073; 95% CI 0.6211–0.7936; *P* < 0.0001

**Fig. 5:**
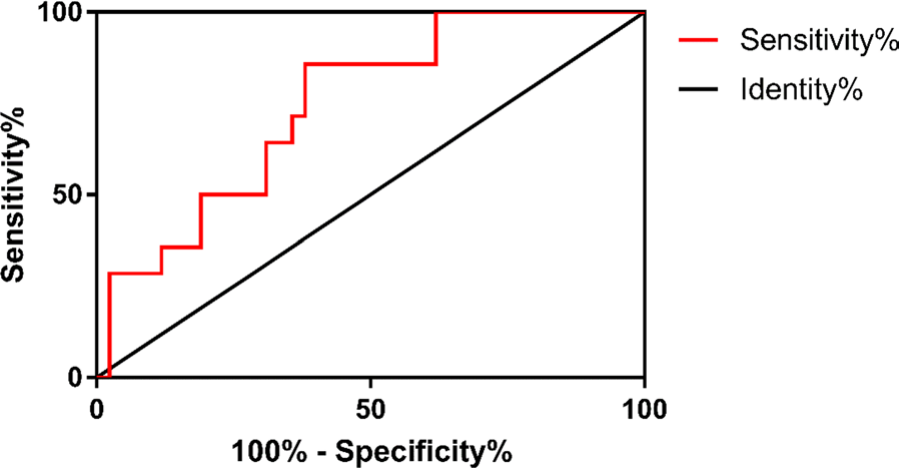
ROC curve of neutrophil counting for acute exacerbation of chronic cholecystitis and acute purulent cholecystitis. The area under curve: 0.7449; 95% CI 0.6087–0.8811; *P* = 0.0064

In terms of the percentage of neutrophil, we found that it may be eligible for the detection of both AGC and APC. While comparing AGC and ACC, we found that the AUC was 0.7073 (95% CI 0.6211–0.7936, *P* < 0.0001) (Fig. 4). With a cut-off value of 79.75%, the sensitivity was 77.92% and the specificity was 61.9%, respectively. The odds ratio (OR) was 5.735 with statistically significant difference (*P* < 0.0001) (Table 4). As for the comparison between APC and ACC, the AUC was 0.7449 (95% CI 0.6087–0.8811) (Fig. 5). With a cut-off value of 80.5%, the sensitivity was 85.71% and the specificity was 61.9%, respectively. The odds ratio (OR) was 9.75 with statistically significant difference (*P* = 0.002) (Table 4). The percentage of neutrophil can be used, therefore, for the rough screening and distinguishing of APC and AGC from ACC as the first step. Afterwards, WBC and GBWT can be applied for the specific detection of AGC and APC, respectively.

## Discussion

Preoperative prediction of severe cholecystitis (SC) is of great significance, as SC is associated with extensive use of antibiotics and longer duration of hospital stay [10]. Early prediction of SC may lead to timely specific therapies, such as early percutaneous cholecystostomy that may effectively reduce the complication rate and the duration of hospital stay of SC patients [11]. SC is associated with more adverse clinical features than ACC. Patients of SC tend to suffer from a variety of complications, such as damage to the main biliary ducts, ligation of aberrant hepatic ducts, and injury to the right hepatic artery during surgery. Once SC is detected before the operation, it is essential for doctors to select appropriate medical measures to avoid related complications and reduce the mortality rate [12, 13]. Imaging techniques, such as abdominal ultrasound and computerized tomography (CT) scanning are routinely used to diagnose ACC. However, it seems that these methods are ineffective for the detection of SC. In the present study, we demonstrated that the application of GBWT, the percentage of neutrophils and the WBC could respectively differentiate APC and AGC from ACC. Therefore, these three indexes could be used to determine surgical priority, improving diagnostic accuracy, and rate, and predicting patients’ risk of progressing from chronic to severe cholecystitis.

In previous studies, neutrophil to lymphocyte ratio (NLR) was widely used to differentiate SC from ACC, as NLR is related to inflammatory responses [14]. Additionally, some indexes of BRE, including ALT, ALP and AST were considered as risk factors affecting cholecystitis-associated mortality [15]. Based on these previous studies, we hypothesized that BRE indexes may contain key information for the diagnosis of different types of cholecystitis. Therefore, we tried to select some indexes from BRE to precisely differentiate two kinds of severe cholecystitis (APC and AGC) from ACC. We detected significant differences between two kinds of SC and ACC by one-way ANOVA. Specifically, APC and ACC significantly differed in terms of GBWT and the percentage of neutrophils, while AGC and ACC differed in terms of ALP, WBC and the percentage of neutrophils. No significant difference was detected for all other indexes. Therefore, GBWT, ALP, the percentage of neutrophils and WBC were selected as our candidate indexes while the rest of indexes including ALT, AST, FIB, GGT, PT and TB were excluded from the analysis.

ROC curve and odds ratio (OR) based on Chi-square test are widely used to evaluate the quality of a prediction model. To evaluate the value of GBWT, ALP, the percentage of neutrophils and WBC for the prediction of three kinds of SC, we created according ROC curves and calculated ORs [16].

As the significant difference in GBWT was only detected between APC and ACC, we believed this index may be optimal for the differentiation of APC from ACC. The analysis based on ROC curve showed that GBWT was effective in the differentiation of APC from ACC with the cut-off value of 5.5 mm, with the GBWT value over 5.5 mm, suggesting the diagnosis of APC. Previous study showed that purulent cholecystitis patients are likely to suffer from severe inflammation and increase of gallbladder drainage [17]. This implies that the gallbladder may be filled with stagnated or purulent contents due to the cholecystitis-induced inflammation and abscess on the gallbladder wall during the cholecystitis. As a result, the GBWT would increase, which is consistent with our findings.

Neutrophils are considered a marker of acute inflammation, as they are recruited to the sites of inflammation [18, 19]. As a result, the percentage of neutrophils would increase in severe inflammation. White blood cells (leukocytes) are also associated with a variety of inflammatory processes. For example, Ryder et al. found that leukocyte counts were associated with inflammation induced by obesity [20]. Bakhtiary et al. discovered a positive correlation between the level of leukocytes and inflammation. Their research showed that the depletion of leukocytes caused the reduction of inflammatory response [21]. Based on these results and our statistical data, we speculated that the percentage of neutrophil and WBC may be used to predict AGC. The analysis based on ROC curves showed that the percentage of neutrophils and WBC were both effective in the differentiations of AGC from ACC with the cut-off values of 79.75% and 11.1*10^9^ /L. The percentage of neutrophils was also effective in differentiating APC from ACC with the cut-off value of 80.5%. To increase the accuracy of our diagnosis, our strategy was to distinguish cases of AGC and APC from ACC roughly using the percentage of neutrophils (cut-off value 79.75%), and then to diagnose AGC and APC using the unique indexes WBC and GBWT, respectively.

Although significant differences were detected between AGC and ACC in the levels of ALP, this index was removed from our study as it was proved to be ineffective to differentiate AGC from ACC, as indicated by the analysis of the ROC curves.

Previous studies always applied a simple index (e.g. NLR, WBC counting) to establish a prediction model for a certain SC, such as AGC [5, 22]. In contrast, we comprehensively applied various BRE indexes and a traditional computed tomography (CT) index, GBWT, to evaluate patients’ situations and precisely differentiated these patients into three categories, including two kinds of severe cholecystitis and acute exacerbation of chronic cholecystitis. This ability to precisely differentiate cholecystitis patients into three specific categories is very important, as it would allow clinicians to select the most suitable treatment options based on the concrete characteristics of each kind of cholecystitis, especially severe cholecystitis.

We report relatively low mortality rate in AGC and APC patients (5.26 and 3.51%, respectively). This can be related to the high conversion rate of laparoscopy in our study that may prevent postoperative complications caused by injury of adjacent organs (such as common bile duct, hepatic artery, portal vein, duodenum, etc.) during the operation. Therefore, in case of complicated gallbladder surgery in the elderly, timely conversion can ensure the safety of surgery and reduce mortality. Patients who died in this article were all emergency operation patients, with an average age of 80 years, and had severe basic diseases (such as lung infection, heart failure, kidney failure, etc.) before the operation.

Moreover, the average hospital stay was 9.32 days. 35.65% of patients stayed in ICU after surgery, with an average of 5.68 days and an average age of 74 years. Among them, 80.49% were emergency patients, and most of them were accompanied by basic diseases (such as hypertension, pulmonary infection, renal insufficiency, etc.) before the surgery.

There are some limitations in our study. Firstly, all patients were from the same hospital, which may decrease the representativeness of cases in our study to some extents. In addition, the number of cases in our study (114) was not sufficient. In particular, sample size of the APC group was only eight patients. Further studies with larger sample sizes are needed to enlarge the number of APC cases to further prove the reliability of GBWT as a predictive index for APC patients. Thirdly, we did not collect data about C reactive protein, an important index which is widely applied to evaluate inflammation [23, 24]. Future studies may include this index to modify our predictive models for cholecystitis.

## Conclusion

In conclusion, we established a predictive model for the differentiations of APC and AGC from ACC with the comprehensive application of the percentage of neutrophils, GBWT and WBC, and set three cut-off values for these indexes: 79.75%, 5.5 mm and 11.1*10^9^ /L, respectively. Once the values of these indexes exceed the cut-off values, patients will be respectively considered as APC and AGC cases. Otherwise, they will be considered as ACC cases. Our results may allow clinicians to improve diagnostic accuracy and further optimize the treatment of severe cholecystitis.

## Data Availability

The datasets generated and analysed during the current study are not publicly available due (It is not convenient to provide datasets because it involves patient privacy), but are available from the corresponding author on reasonable request.

## Abbreviations

SC: Severe cholecystitis
AGC: Acute gangrenous cholecystitis
APC: Acute purulent cholecystitis
ACC: Acute exacerbation of chronic cholecystitis
ICU: Intensive care unit
WBC: White blood cell
BRE: Blood routine examination
GBWT: Gallbladder wall thickness
